# Influenza A (H1N1) in Rome, Italy in family: three case reports

**DOI:** 10.1186/1757-1626-2-9123

**Published:** 2009-12-01

**Authors:** Francesco Lisena, Licia Bordi, Fabrizio Carletti, Concetta Castilletti, Federica Ferraro, Eleonora Lalle, Simone Lanini, Luca Enrico Ruscitti, Francesco Maria Fusco

**Affiliations:** 1 Istituto Nazionale per le Malattie Infettive IRCCS Lazzaro Spallanzani - Roma Via Portuense, 292 - 00149 Rome, Italy

## Abstract

**Introduction:**

A new Influenza A virus H1N1 appeared in March-April 2009, and thousands of cases are being reported worldwide. In the initial months, several imported cases were reported in many European countries, while some countries reported local chains of transmission. We describe the first cluster of in-country transmission of the new Influenza A H1N1 which occurred in Italy, involving 3 patients.

**Case presentation:**

Patient 1, a 11-year-old male child developed fever, cough, and general malaise 4 days after returning from a travel to Mexico. Some days later, the 69-year-old grandfather (patient 2), who did not travel to Mexico, and the 33-month-old brother (patient 3) of patient 1 developed mild influenza symptoms. PCR tests resulted positive for Influenza A, and sequence analysis confirmed infection with the Influenza A (H1N1) strain for all three patients. Some problems were experienced in the administration of chemoprophylaxis and therapy in the patient 3. The chemoprophylaxis policies in other family members are described, too.

**Conclusion:**

Some interesting facts emerge from the analysis of this cluster. The transmission of Influenza A H1N1 virus seems to be dependent on strict contacts. Patient 2 and patient 3 did not take the chemoprophylaxis properly. The problems in the administration of chemoprophylaxis and therapy to patient 3 suggest that in infants specific individual-based strategies for assuring the correct administration are advisable.

## Background

Swine Influenza virus has in the past caused sporadic infections in humans, causing different clinical pictures ranging from mild respiratory symptoms to, rarely, severe diseases with pneumonia and deaths [1]. A new Influenza A H1N1 virus of swine origin appeared in March-April 2009, and because of its capability to be directly transmitted from human to human, thousands of cases are now being reported worldwide. In the initial months, in Europe many imported cases were reported. Sustained autochthonous transmission firstly appeared in Spain and United Kingdom [2], while presently local chains of transmission are ongoing in most European countries. Hereby we describe a familiar cluster involving 3 patients. This is the first in-country transmission of the new Influenza A H1N1 described in Italy.

## Case presentation

### Patient 1

On May 3, patient 1, a 11-year-old male child presented for medical evaluation at Admission Department of the National Institute for Infectious Disease (Istituto Nazionale per le Malattie Infettive, INMI) "Lazzaro Spallanzani", in Rome, Italy.

He reported abrupt onset of fever, cough, and general malaise in the night between 2^nd ^and 3^rd ^of May. He had returned on April 29, with his parents, a sister and a brother, from Mexico, in the Yucatan area.

At presentation the patient was febrile (38°C), and reported coryza and bilateral pain in the ear. According to case definition in use at our Institute the patient was managed as a suspected case of Influenza A H1N1. A nasopharyngeal swab was taken, and a rapid test was positive for Influenza A. Subsequently, a PCR test was positive for Influenza A, and sequence analysis confirmed infection with the new Influenza A (H1N1) strain. A chest X-ray did not show any evidence of pneumonia. On the same day therapy with oseltamivir was started, at dosages appropriate for the patient's weight, then the patient was transferred to a referral paediatric hospital, where he was isolated. The patient completely recovered in few days, and was discharged after 7 days of isolation.

### Patient 2

On May 5, patient 2, a 69-year-old man, grandfather of patient 1, referred to INMI Admission Department, because of onset of fever (37,4°C), the day before, on May 4. At history taking he reported to have had close contact with his nephew (patient 1).

At the admission visit, general conditions of patient 2 were good, and the patient was already afebrile. The patient was coughing forcefully, although he reported it was a usual symptom being a smoker.

The rapid test for Influenza A was negative. Subsequently, the PCR was reported positive for Influenza A, and sequence analysis confirmed infection with the same strain affecting patient 1. Patient 2 refused hospital admission, then treatment with oseltamivir was started, and the patient was quarantined at home for 7 days. His general condition improved, and he did not have fever or other symptoms thereafter.

### Patient 3

On May 6, patient 3, a 33-month-old infant, brother of patient 1, was accompanied by his father for evaluation to the referral paediatric hospital where the patient 1 had been isolated. He also had fever (38,2°C) and coryza, and the father reported that the infant was irritable and unwell. A rapid test for Influenza was positive, and the patient was sent home with advise to take treatment with oseltamivir. Since May 4, Patient 3 was on chemo-prophylaxis with oseltamivir, but adherence was poor because, despite the use of the syrup, the child refused to swallow and spew out the drug.

On May 7, the father and the patient 3 came for medical examination and advice to INMI. At the admission visit, only mild coryza was recorded. A second rapid test for Influenza resulted negative, while the PCR resulted positive for Influenza A. The switch to therapeutic dosage of oseltamivir was confirmed, and physicians also suggested administering the drug with some sweet foods or beverages. In such a way, the patient 3 had therapy regularly, and no more symptoms were noticed. Sequence analysis confirmed the infection with the new strain of Influenza A H1N1 virus.

### Other family members

The mother of patients 1 and 3, who gave care to patient 1 during his admission to paediatric hospital, started prophylaxis with oseltamivir on May 3. The 14-year-old sister of patients 1 and 3 began the prophylaxis with oseltamivir on May 4. The grandmother, who slept together with patient 1 at the moment of symptoms' onset, and the father of patients 1 and 3 started prophylaxis on May 5. None of them developed symptoms.

### Laboratory analysis

*Rapid Influenza testing *at INMI was based on BinaxNOW Influenza A & B, able to detect and differentiate Influenza A and B. The test was performed on freshly collected nasopharyngeal swab samples, immediately upon arrival to the laboratory.

*Nucleic acid extraction*: nasopharyngeal swab samples were extracted with MagMAX TM- 96 viral RNA Isolation Kit (Ambion, Applied Biosystem), according to manufacturer's instructions, using MagMAX TM Express magnetic particle processor. The efficiency of the extraction was evaluated using a method previously established in our laboratory [3].

*Molecular testing*: the detection of Influenza A was based on RT-PCR, using a primer set with enhanced sensitivity for the new Influenza A H1N1 strain, targeting NP (Nucleo Proteins), established in our laboratory on the basis of the sequences of swine AH1N1 published on the GSAID (Global Initiative on Sharing Avian Influenza Data) Web site. The detailed protocol will be described elsewhere (Di Caro et al., manuscript in preparation).

All the RT-PCR reactions were performed using One-Step RT-PCR Enzyme Mix (Qiagen, Valencia, CA).

Positive results for the new Influenza A H1N1 were confirmed by the National Reference Laboratory for Influenza.

The strain identification was based on the sequence analysis of the amplicon (282 bp), performed with the automated ABI Prism 3100 instrument, by using BigDye terminator cycle sequencing kit (Applied Biosystem, Warrington, UK). The primers used for the sequencing reaction were the same as those used for the RT-PCR. The NP amplicon sequences of the 3 patients were compared to GenBank reference sequences by BLAST (Basic Local Alignment Search Tool) search analysis. In all cases, the highest hit were new Influenza A H1N1 strains, and Blast search analysis results indicated 98, 99 and 99% (respectively, for patients 1, 2 and 3) identity over the corresponding stretch of 282 nucleotides sequence of the California 04/2009 H1N1 isolate from the current outbreak (GenBank accession number FJ969512).

All the NP partial sequences have been submitted to GenBank, accession numbers FJ985805, GQ132135, GQ132187.

## Conclusion

This report briefly describe the first cluster of in-country transmission of the new Influenza A H1N1 which occurred in Italy.

**Figure 1:**
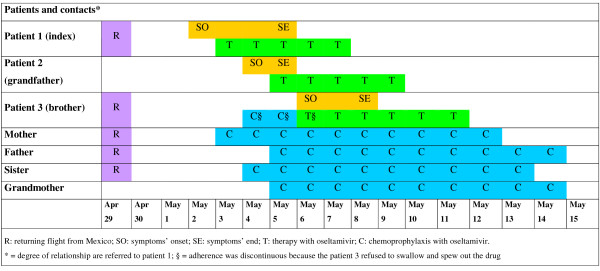
**Timeline of a familiar outbreak of new Influenza A H1N1 in Italy**.

The figure 1 summarized the timeline of this described cluster. The index patient, patient 1, most probably acquired the infection during the travel, with his family, in Mexico. Patient 2 did not visit the endemic areas, and presumably acquired the infection from the nephew (patient 1), who slept with him during the night when symptoms had appeared. On the basis of time lapse between return from Mexico and start of symptoms (8 days), it is likely that patient 3 also acquired the infection in Italy, following close contacts with his brother (patient 1), or, alternatively, from his grandfather (patient 2). Indeed, patients with the new Influenza A H1N1 are assumed to be shedding virus from one day prior to illness onset until resolution of symptoms [4]. In the case he acquired the infection from the grandfather, patient 3 may represent a tertiary case. Since children are likely to have a social behaviour prone to cause close contacts with secretions, it is reasonable that he had had high-risk exposures to the secretions of the brother or of the grandfather.

Notably, patient 2 and patient 3 did not assume or assumed not properly the chemoprophylaxis, respectively. The remaining close contacts who assumed chemoprophylaxis promptly (the mother and the sister of patients 1 and 3, who respectively started chemoprophylaxis on May 3 and 4), or later (the grandmother and the father of patients 1 and 3) did not develop any symptoms, although viral transmission cannot be ruled out in absence of laboratory tests (i.e. seroconversion).

At the beginning of the pandemic, when these patients were observed, the treatment with oseltamivir (75-mg capsule twice per day for 5 days for adults, weight-based for children) or zanamivir (two 5-mg inhalations -10 mg total - twice per day for 5 days for adults and children older than 7-year-old) was usually administered to all patients. For this reason, in the described cluster therapy has been given also to those patients presenting with mild symptoms, as patients 2 and 3.

Currently, the use of antiviral therapy is suggested in suspected/probable/confirmed patients, with priority given to hospitalized patients and patients at higher risk for influenza complications. Antiviral treatment with zanamivir or oseltamivir should be initiated as soon as possible after the onset of symptoms [5]. Oseltamivir is administered orally, and zanamivir by inhalation, but the successful use of intravenous zanamivir in a patient with severe H1N1 pneumonitis has been recently reported [6]

Chemoprophylaxis with the same drugs (oseltamivir 75-mg capsule once per day for 10 days for adults, weight-based for children, and zanamivir, two 5-mg inhalations - 10 mg total - once per day for 10 days in adults and children older than 5-year-old) is currently recommended only to those persons at high risk for complications of influenza [5]. At the beginning of the pandemic, instead, chemoprophylaxis was usually suggested to all close contacts of the patients with new Influenza A H1N1.

The management of the infant presents interesting issues: indeed, in this patient the administration of chemoprophylaxis and therapy required special care. In paediatric patients, specific individual-based strategies must be developed in order to assure the regular administration of oseltamivir.

All described cases were mild, and promptly recovered in few days. Similar clinical pictures are reported in the vast majority of other cases in the world.

Our observation also evidences the poor sensitivity of rapid diagnostic tests for Influenza, as confirmed by a recent study [7]. In our three confirmed patients, four rapid tests were performed. It was positive only in patient 1, while resulted negative in patient 2. In patient 3, rapid test was positive on the day of symptoms' onset, but rapidly turned negative on day after.

## Consent

Written informed consent was obtained from the patients for publication of this case report. A copy of the written consent is available for review by the Editor-in-Chief of this journal.

## Competing interests

The authors declare that they have no competing interests.

## Authors' contributions

FMF designed the study, wrote and drafted the manuscript; LB, FC, CC and EL performed virological and molecular tests and experiments, and revised critically the manuscript; FL, FF, SL and LER collected, assembled and analyzed the data, and revised critically the manuscript. All authors read and gave final approval of the version to be published.
